# Assessment of Myocardial Microstructure in a Murine Model of Obesity-Related Cardiac Dysfunction by Diffusion Tensor Magnetic Resonance Imaging at 7T

**DOI:** 10.3389/fcvm.2022.839714

**Published:** 2022-04-05

**Authors:** David Lohr, Arne Thiele, Max Stahnke, Vera Braun, Elia Smeir, Joachim Spranger, Sebastian Brachs, Robert Klopfleisch, Anna Foryst-Ludwig, Laura M. Schreiber, Ulrich Kintscher, Niklas Beyhoff

**Affiliations:** ^1^Chair of Molecular and Cellular Imaging, Comprehensive Heart Failure Center (CHFC), University Hospital Wuerzburg, Wuerzburg, Germany; ^2^Charité – Universitätsmedizin Berlin, Corporate Member of Freie Universität Berlin and Humboldt-Universität zu Berlin, Institute of Pharmacology, Cardiovascular-Metabolic-Renal Research Center, Berlin, Germany; ^3^German Centre for Cardiovascular Research (DZHK), Partner Site Berlin, Berlin, Germany; ^4^Charité – Universitätsmedizin Berlin, Corporate Member of Freie Universität Berlin and Humboldt-Universität zu Berlin, Department of Endocrinology and Metabolism, Cardiovascular-Metabolic-Renal Research Center, Berlin, Germany; ^5^Department of Veterinary Pathology, College of Veterinary Medicine, Freie Universität Berlin, Berlin, Germany; ^6^Berlin Institute of Health at Charité – Universitätsmedizin Berlin, Berlin, Germany; ^7^Charité – Universitätsmedizin Berlin, Corporate Member of Freie Universität Berlin and Humboldt-Universität zu Berlin, Department of Cardiology, Campus Benjamin Franklin, Berlin, Germany

**Keywords:** cardiac adipose tissue, cardiac dysfunction, diffusion tensor imaging, magnetic resonance imaging, obesity, high-fat diet, obesity-related cardiac dysfunction

## Abstract

**Background:**

Obesity exerts multiple deleterious effects on the heart that may ultimately lead to cardiac failure. This study sought to characterize myocardial microstructure and function in an experimental model of obesity-related cardiac dysfunction.

**Methods:**

Male C57BL/6N mice were fed either a high-fat diet (HFD; 60 kcal% fat, *n* = 12) or standard control diet (9 kcal% fat, *n* = 10) for 15 weeks. At the end of the study period, cardiac function was assessed by ultra-high frequency echocardiography, and hearts were processed for further analyses. The three-dimensional myocardial microstructure was examined *ex vivo* at a spatial resolution of 100 × 100 × 100 μm^3^ by diffusion tensor magnetic resonance imaging (DT-MRI) at 7T. Myocardial deformation, diffusion metrics and fiber tract geometry were analyzed with respect to the different myocardial layers (subendocardium/subepicardium) and segments (base/mid-cavity/apex). Results were correlated with blood sample analyses, histopathology, and gene expression data.

**Results:**

HFD feeding induced significantly increased body weight combined with a pronounced accumulation of visceral fat (body weight 42.3 ± 5.7 vs. 31.5 ± 2.2 g, body weight change 73.7 ± 14.8 vs. 31.1 ± 6.6%, both *P* < 0.001). Obese mice showed signs of diastolic dysfunction, whereas left-ventricular ejection fraction and fractional shortening remained unchanged (E/e’ 41.6 ± 16.6 vs. 24.8 ± 6.0, *P* < 0.01; isovolumic relaxation time 19 ± 4 vs. 14 ± 4 ms, *P* < 0.05). Additionally, global longitudinal strain was reduced in the HFD group (−15.1 ± 3.0 vs. −20.0 ± 4.6%, *P* = 0.01), which was mainly driven by an impairment in basal segments. However, histopathology and gene expression analyses revealed no myocardial fibrosis or differences in cardiomyocyte morphology. Mean diffusivity and eigenvalues of the diffusion tensor were lower in the basal subepicardium of obese mice as assessed by DT-MRI (*P* < 0.05). The three-dimensional fiber tract arrangement of the left ventricle (LV) remained preserved.

**Conclusion:**

Fifteen weeks of high-fat diet induced alterations in myocardial diffusion properties in mice, whereas no remodeling of the three-dimensional myofiber arrangement of the LV was observed. Obese mice showed reduced longitudinal strain and lower mean diffusivity predominantly in the left-ventricular base, and further investigation into the significance of this regional pattern is required.

## Introduction

Obesity is a major risk factor for heart failure (HF) with rising prevalence worldwide during the past ∼50 years (1, 2). It is now recognized that obesity represents a chronic progressive disease clearly distinct from only being a risk factor for other conditions that predispose for HF development like type 2 diabetes or hypertension (3). This conception is supported by recent evidence indicating that the link between obesity and HF is stronger than those for other cardiovascular diseases and insufficiently explained by traditional risk factors (4). Furthermore, obesity-related HF with a preserved ejection fraction (HFpEF) is considered as a genuine form of cardiac failure as it is associated with clinical characteristics that differ substantially from other HF phenotypes (5).

Excessive adiposity causes structural and functional cardiac alterations that ultimately may lead to HF (6, 7). Obese individuals have higher amounts of myocardial fat content and cardiac adipose tissue, which have been linked to reduced myocardial contractile function (8). Cardiovascular imaging studies showed that left-ventricular impairment is already present in asymptomatic obese individuals and that these changes can be detected even as early as during childhood and adolescence (6, 9). However, data on morphologic-functional relationships in obesity-related cardiac dysfunction is scarce and the impact of excessive body fat accumulation on the three-dimensional myofiber architecture of the heart remains unknown.

Cardiac diffusion tensor magnetic resonance imaging (DT-MRI) is a unique method to quantify structure, organization and integrity of the myocardium at the microscopic scale (10). Nevertheless, the value of cardiac DT-MRI in obesity-related cardiac dysfunction is uncertain.

In the present study, the cardiac phenotype of a mouse model of diet-induced obesity was comprehensively characterized by DT-MRI, ultra-high frequency echocardiography, and histopathology regarding myocardial microstructure and function. We hypothesized that (1) high-fat diet (HFD) feeding in mice induces obesity-related cardiac dysfunction; and (2) diet-induced obesity is accompanied by altered cardiac diffusion properties as well as remodeling of the three-dimensional myofiber arrangement of the left ventricle (LV).

## Materials and Methods

All *in vivo* experiments were carried out in accordance with the German Law and directive 2010/63/EU of the European Parliament on the protection of animals used for scientific purposes. The study protocol was prospectively prepared and approved by local authorities (G0226/19, Landesamt für Gesundheit und Soziales Berlin, Germany). Results are reported according to the ARRIVE guidelines.

### Study Protocol

**FIGURE 1:**
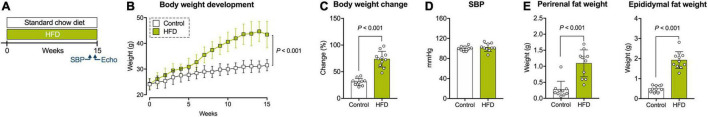
Study protocol and physiological measures. **(A)** Study protocol. **(B)** Body weight development during the study period. **(C)** Relative body weight change from baseline to final assessment. **(D)** Systolic blood pressure (SBP) at 14 weeks of dietary intervention. **(E)** Weight of perirenal and epididymal fat depots after 15 weeks of dietary intervention. Echo, echocardiography; SBP, systolic blood pressure. Mean ± SD; *n* = 10–11 per group; two-way repeated measures ANOVA **(B)**, unpaired Student’s *t*-test (**C,E**, right panel), or Mann-Whitney *U* test (**E**, left panel).

The study protocol is illustrated in Figure 1A. Male C57BL/6N mice (8–9 weeks old) were obtained from Janvier Labs (France) and maintained group-housed in environmentally controlled, individually ventilated cages (21.5 ± 1.5°C) with a 12-h light/dark cycle and unrestricted access to food and water. Animals were randomized according to baseline body weight to receive either a HFD (60 kcal% fat; diet formula D12492, Research Diets, United States; *n* = 12) or standard control diet (9 kcal% fat; ssniff Spezialdiäten, Germany; *n* = 10) for 15 weeks. Weight development and food consumption were monitored twice a week. After 14 weeks of dietary intervention, blood pressure measurements were performed. Cardiac function was assessed by transthoracic echocardiography before the animals were sacrificed after 15 weeks of dietary intervention via cervical dislocation under inhalational anesthesia with isoflurane. Organs and visceral fat depots were excised and weighed. The right superior lung lobe was weighed immediately after removal (wet weight) and again after being dried to a constant weight (dry weight) for calculation of the wet-to-dry lung weight ratio. The mean tibia length was used to normalize organ weights.

### Blood Pressure Measurements

Systolic blood pressure was measured non-invasively in conscious animals using a mouse tail cuff method blood pressure system (IITC Life Science, United States). Briefly, animals were restrained and placed in a dedicated chamber with temperature being maintained at 37°C. After ∼5 min of acclimatization, a minimum of three different measurements were obtained and averaged per animal. To reduce stress during final measurements, animals were trained by exposing them to the procedure several times before.

### Echocardiography

Mice were anesthetized by isoflurane inhalation and placed in supine position on a heated examination table with integrated ECG electrodes (37°C). The chest was depilated and prewarmed ultrasound gel was applied. Transthoracic echocardiography was performed using an ultra-high frequency linear transducer (30 MHz; MX400) coupled to a Vevo 3100 Imaging System (both FUJIFILM VisualSonics, Canada) as described before (11). Acquired images were analyzed with a dedicated software package (Vevo LAB, FUJIFILM VisualSonics).

Left-ventricular ejection fraction was derived from the parasternal long axis view using a modified Simpson’s method of disks. End-diastolic wall thickness and fractional shortening of the LV were measured in M-Mode at mid-papillary level in the parasternal short axis view. Mitral flow velocities were assessed with pulsed-wave Doppler above the mitral leaflets; tissue velocities were obtained with tissue Doppler at the medial mitral annulus.

B-Mode images were processed with a dedicated software algorithm for myocardial strain analyses (Vevo Strain, FUJIFILM VisualSonics). Longitudinal strain and strain rate were derived from the parasternal long axis view, whereas radial and circumferential parameters were generated from the parasternal short axis view. Three different tracings of the endocardial and epicardial border were performed, and all parameters were averaged over three consecutive cardiac cycles. Longitudinal deformation was assessed along both the endocardial and epicardial border; circumferential parameters were acquired from endocardial tracings only. Radial parameters were derived by analysis of the deformation of endocardial points and corresponding ones along the epicardial border. In addition, longitudinal layer-specific strain and -strain rate were assessed along the endocardial and epicardial border. The myocardium was divided into six segments, and segmental strain curves were averaged for calculation of global strain and strain rate parameters. Segmental longitudinal strain values were calculated as the mean of the two apical, mid-cavity and basal segments, respectively. Strain was indicated as peak systolic strain; longitudinal, radial and circumferential strain rates were given as peak systolic strain rate (SR_*sys*_). Analysis of longitudinal deformation also included assessment of peak diastolic strain rate (longitudinal SR_*dia*_).

### Blood Sample Analyses

Blood samples were collected via puncture of the retrobulbar venous plexus under anesthesia with isoflurane immediately before necropsy. A random blood glucose test was performed using a clinical glucose meter (Contour XT, Bayer, Germany). Subsequently, samples were centrifuged, and serum aliquots were stored at −80°C until further measurements. Circulating levels of TIMP-1 (tissue inhibitors of metalloproteinases 1) were quantified by an enzyme-linked immunosorbent assay according to the manufacturer’s instructions (R&D Systems, United States). All other parameters were determined by colorimetry using an AU480 clinical chemistry analyzer (Beckman Coulter, United States).

### Heart Preparation

A subset of heart specimens was prepared for DT-MRI (*n* = 5 per group), whereas the remaining samples were processed for further tissue analyses. Preparation for DT-MRI was performed as previously described (11). Briefly, the heart was excised, and extracardiac fat tissue was removed. Afterward, the ascending aorta was dissected free and cannulated for retrograde perfusion with cardioplegic solution (20 mM potassium chloride in cold phosphate-buffered saline). Following perfusion fixation and storage in 4% formalin for exactly 28 days, samples were sent to the MRI site. Hearts designated for DT-MRI were excluded from gravimetric analyses as their weights were not comparable due to the outlined preparation procedure.

### Diffusion Tensor Magnetic Resonance Imaging

High resolution DT-MRI was performed at the Comprehensive Heart Failure Center, University Hospital Würzburg, Germany. Prior to MRI measurements, hearts were rinsed using saline and then placed in a 2 ml syringe. Hearts were surrounded with the susceptibility matching medium Fomblin™ (Solvay Specialty Polymers, Italy) in order to prevent distortions at tissue-medium interfaces and to adjust the receive chain of the MR system to signal from heart tissue. Prior to sealing the syringe, a dull needle was used to carefully compress both ventricles in order to remove any remaining saline solution or trapped air. Syringes were fixed on a dedicated 3D-printed sledge that enabled consistent placement with respect to the scanner isocenter and the radiofrequency coil. Hearts remained in Fomblin™ for the duration of the measurement (∼12 h) and were placed in phosphate-buffered saline afterward.

All MRI measurements were performed using a PharmaScan™ (70/16) 7T MRI system and a two-channel ^1^H-cryoprobe (all Bruker BioSpin, Germany). Axial, coronal, and sagittal gradient echo acquisitions with 50 μm in-plane resolution and a slice thickness of 0.4 mm were used to determine the short axis orientation. Echo times of 3.3 ms allowed to detect image distortions caused by trapped air. DT-MRI data were acquired for 90 slices with 100 μm isotropic resolution using a spin echo sequence with standard readout and monopolar diffusion encoding (2.5 ms gradient duration and 8.4 ms gradient separation). Two reference images (*b* = 0 s/mm^2^) were acquired, while the signal attenuation induced by the diffusion process was measured in 12 directions (b_*max*_ = 933 s/mm^2^). Further measurement parameters were TE/TR: 17.5/4000 ms, bandwidth: 447 Hz/Pixel, field of view: 10 mm × 10 mm, matrix size: 100 × 100 resulting in an in-plane spatial resolution of 0.1 mm × 0.1 mm. Total scan time for 10 averages of the DT-MRI protocol amounted to 11 h and 40 min.

DT-MRI images were denoised using over complete local partial component analysis (12) and then segmented according to the 17-segment model of the AHA. Based on this segmentation we created profiles from endocardium to epicardium, connecting voxels on the endocardial contour to the closest voxel on the epicardial contour. Profile distances were used to calculate the wall thickness and to split the myocardium along the center, creating a subendocardial and a subepicardial layer for the assessment of diffusion and microstructure metrics. We established a local orthogonal coordinate system with longitudinal, circumferential, and radial axes for every voxel within the myocardium. Projections of the primary and secondary eigenvector of diffusion within this coordinate system were used to determine helix and sheetlet angle values as described before (13). Transmural helix angle profiles were created by calculating average values at 0, 25, 50, 75, and 100% of the left-ventricular wall. With the exception of image denoising, all post processing was done using in-house developed MATLAB (MathWorks, United States) code and DSI Studio^1^ (15 November 2018 build). All images depicting tractography of cardiomyocyte bundles were generated using fiber tracking algorithms and visualization tools of DSI Studio. Adjustments to interface scripts connecting MATLAB and DSI studio enabled the introduction of helix and sheetlet angle values into the DSI data format. In order to assess fiber bundle coherence on smaller scales, we performed tractography in the LV using a minimal fiber bundle length of 0.1 and 1 mm as termination criteria. Seed points (10,000) were placed within LV segmentation, and a fractional anisotropy of 0.075 as well as angular coherence was required along tracts.

### Histopathology

Cardiac cross sections derived from the cardiac base, mid-cavity and apex were fixed with 4% formalin and paraffin-embedded for staining with hematoxylin/eosin (Carl Roth, Germany) and Picrosirius Red (Morphisto, Germany). A blinded expert in veterinary pathology (RK) was asked to grade cellular damage in slides stained with hematoxylin/eosin using a semiquantitative scale ranging from 0 (no damage) to 3 (severe damage). Similarly, cardiomyocyte hypertrophy was graded on a semiquantitative scale as follows: 0 (all cardiomyocytes with regular size in transverse sections), 1 (few cardiomyocytes with increased size in transverse sections), 2 (some cardiomyocytes with increased size in transverse sections), and 3 (majority of cardiomyocytes with increased size in transverse sections). For collagen quantification, histological slides were digitized using an Aperio CS2 image capture device (Leica Biosystems, Germany), and the relative proportion of red-stained collagen from total tissue area was determined by a software algorithm (Aperio ImageScope and Aperio GENIE, both Leica Biosystems). Total collagen in the LV was calculated as the mean of apical, mid-cavity and basal values.

### Quantitative Real-Time PCR

Total RNA was isolated from frozen cardiac tissue using QIAzol lysis reagent and the RNeasy Micro kit according to the manufacturer’s protocol (Qiagen, Germany). RNA was transcribed into cDNA by reverse transcriptase in combination with ribonuclease inhibitor and deoxynucleotide triphosphates (all Promega, United States). Primer sequences are depicted in Supplementary Table 1. A CFX Connect Real-Time PCR Detection System (Bio-Rad, United States) was used for quantitative PCR. Beta-actin served as an endogenous control and was used to calculate relative expression levels by the 2^–ΔΔ*CT*^ method.

### Statistics

Data are reported as mean ± SD or median [95% confidence interval]. Normality was assessed by Q-Q plots and Shapiro-Wilk test. Differences between the two diet groups were compared by two-tailed unpaired Student’s *t*-test or two-tailed Mann-Whitney *U* test, as appropriate. Body weight development was analyzed by two-way repeated measures ANOVA. A value of *P* < 0.05 was considered statistically significant. Statistical analyses were performed using GraphPad PRISM 7 (GraphPad Software, United States). DT-MRI, echocardiography and histopathology were performed in a blinded manner regarding the experimental group.

## Results

All animals completed the study protocol without mortality or any adverse events. One animal in the HFD group exhibited insufficient body weight gain (< 30%) and was therefore excluded from further analyses (proportion of non-responders: 8.4%).

### Physiological Data

Mice in the HFD group began to weigh significantly more than control animals after 4 weeks of dietary intervention (Figure 1B). Overall, weight gain during the study period was 73.7 ± 14.8% in the HFD group, compared to 31.1 ± 6.6% in the control group (Figure 1C). Systolic blood pressure at 14 weeks of dietary exposure was comparable between both treatments (Figure 1D). Similarly, a random blood glucose measurement at the end of the study period revealed no significant group differences (Table 1). Mice on HFD had higher amounts of circulating levels of total, low-density lipoprotein (LDL), and high-density lipoprotein (HDL) cholesterol, whereas triglycerides were comparable between both diets (Table 1). The LDL/HDL ratio was elevated in obese mice but failed to reach statistical significance (Table 1). No differences were found in CK-MB (creatinine kinase myocardial band) levels as a marker of potential myocardial myofiber damage (Table 1).

**TABLE 1:** Blood sample analyses.

Parameter, unit	Control	HFD	*P* value
CK-MB, U/l	60.4 [48.9–92.7]	60.2 [50.9–87.0]	0.85
Glucose, mg/dl	149.5 [110–190]	155 [138–170]	0.89
HDL, mg/dl	69 [57.7–71.4]	122.7 [95.0–148.4]	**<0.001**
LDL, mg/dl	14.2 [11.0–16.9]	35.6 [23.4–47.0]	**<0.001**
LDL/HDL ratio	0.21 [0.19–0.26]	0.27 [0.21–0.37]	0.08
TIMP-1, pg/ml	1446.0 [906.7–1733.0]	1863.0 [1250.0–2243.0]	0.17
Total cholesterol, mg/dl	101.7 [88.8–111.3]	184.9 [145.0–252.3]	**<0.001**
Triglycerides, mg/dl	117.7 [61.8–208.5]	119.9 [81.2–137.9]	0.49

*CK-MB, creatine kinase myocardial band; TIMP-1, tissue inhibitors of metalloproteinases 1. Median [95% confidence interval]; n = 9–11 per group; unpaired Student’s t-test or Mann-Whitney U test. Bold indicates statistical significance.*

### Necropsy and Myocardial Tissue Analyses

The HFD group showed an excessive accumulation of visceral fat indicated by significantly increased perirenal and epididymal fat depots (Figure 1E). Gross pathology revealed hepatic lipid accumulation in 6/11 animals of the obese group, without significant differences in liver weight (data not shown).

**FIGURE 2:**
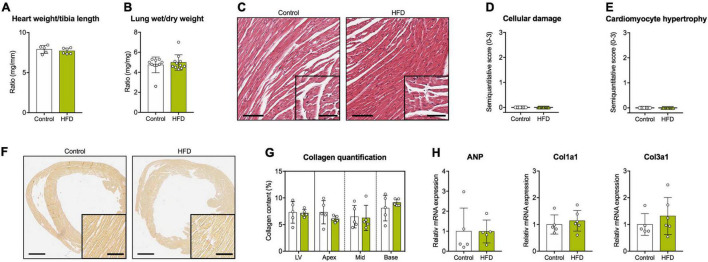
Cardiovascular tissue analyses. **(A)** Heart weight normalized to tibia length. **(B)** Wet-to-dry lung weight ratio. **(C)** Representative cardiac cross sections stained with hematoxylin/eosin (scale bars = 100 μm and 50 μm, respectively). Semiquantitative histological scores for **(D)** cellular damage and **(E)** cardiomyocyte hypertrophy. **(F)** Representative cardiac cross sections stained with Picrosirius Red for the detection of collagen fibers (scale bars = 1 mm and 100 μm, respectively). **(G)** Quantification of myocardial collagen content in the whole LV and the different myocardial segments. **(H)** Gene expression analysis of *ANP*, *Col1a1*, and *Col3a1* in myocardial tissue. *ANP*, atrial natriuretic peptide; *Col1a1*, collagen type I alpha 1 chain; *Col3a1*, collagen type III alpha 1 chain. Mean ± SD; *n* = 5–6 **(A,G,H)** and 10–11 **(B,D,E)** per group; unpaired Student’s *t*-test or Mann-Whitney *U* test (no significant differences were found).

Normalized heart weights did not differ between both groups (Figure 2A). The wet-to-dry lung weight ratio as an index for pulmonary edema was also comparable (Figure 2B).

Histopathology showed unremarkable cardiomyocyte morphology without evidence of relevant cellular damage or hypertrophy (Figures 2C–E). Moreover, no myocardial fibrosis was observed in any diet (Figures 2F,G). These results were in accordance with myocardial gene expression data and blood sample analyses indicating no group differences regarding various markers of extracellular matrix turnover, cardiomyocyte hypertrophy or myofiber damage (Figure 2H and Table 1).

### Cardiac Function Analysis

Echocardiography data acquired at the end of the study period is depicted in Table 2. LV geometry and systolic function, assessed by ejection fraction and fractional shortening, were comparable among both groups. Obese mice showed signs of diastolic dysfunction with prolonged isovolumic relaxation time, reduced early diastolic mitral annular velocity (e’), and an increased ratio of transmitral early filling rate to early diastolic mitral annular velocity (E/e’). Early and late diastolic filling rates (E and A, respectively) were proportionally reduced in HFD animals resulting in unaltered E/A ratios.

**TABLE 2:** Echocardiography data.

Parameter, unit	Control	HFD	*P* value
Heart rate, 1/min	482 ± 34	459 ± 42	0.19
**Parasternal long axis view**			
EF,%	52 ± 5	54 ± 8	0.43
EDV, μl	70 ± 13	64 ± 12	0.25
ESV, μl	35 ± 9	29 ± 8	0.19
**Parasternal short axis view**			
FS,%	28 ± 5	30 ± 9	0.53
LVAW, mm	0.8 ± 0.1	0.8 ± 0.1	0.92
LVPW, mm	0.7 ± 0.1	0.7 ± 0.2	0.42
**Apical four chamber view**			
E, mm/s	692 ± 120	596 ± 67	**0.04**
A, mm/s	448 ± 92	381 ± 50	** < 0.05**
e’, mm/s	29 ± 4	16 ± 6	** < 0.001**
a’, mm/s	25 ± 5	21 ± 4	0.052
E/A	1.6 ± 0.3	1.6 ± 0.2	1.00
E/e’	24.8 ± 6.0	41.6 ± 16.6	**0.007**
e’/a’	1.2 ± 0.3	0.9 ± 0.4	0.06
IVRT, ms	14 ± 4	19 ± 4	**0.006**
IVCT, ms	14 ± 3	15 ± 5	0.78
**Speckle-tracking echocardiography**			
Global radial strain,%	35.1 ± 6.0	35.0 ± 13.2	0.97
Global radial SR_*sys*_, 1/s	8.8 ± 1.8	8.7 ± 3.2	0.47
Global circumferential Strain,%	−20.6 ± 3.7	−18.7 ± 3.8	0.25
Global circumferential SR_*sys*_, 1/s	−7.1 ± 1.9	−6.7 ± 2.4	0.28

*A, late diastolic filling rate; a’, late diastolic mitral annular velocity; E, early diastolic filling rate; e’, early diastolic mitral annular velocity; EF, ejection fraction; FS, fractional shortening; EDV, end-diastolic volume; ESV, end-systolic volume; IVRT, isovolumic relaxation time; and IVCT, isovolumic contraction time; LVAW, left-ventricular anterior wall thickness; LVPW, left-ventricular posterior wall thickness. Mean ± SD; n = 10–11 per group; unpaired Student’s t-test or Mann-Whitney U test. Bold indicates statistical significance.*

**FIGURE 3:**
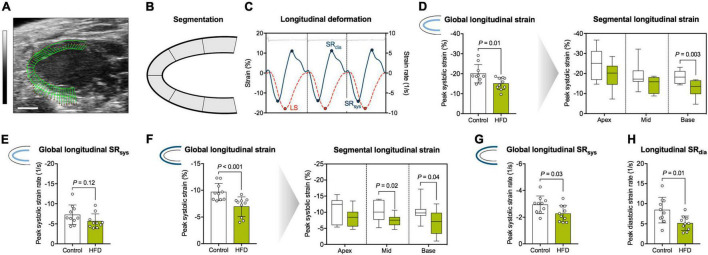
Analysis of myocardial longitudinal deformation. **(A)** Representative B-Mode image in the parasternal long axis view used for speckle-tracking analysis (scale bar = 2 mm; vector arrows along the endocardial border). **(B)** The myocardium was divided into six segments, and longitudinal strain was assessed as their average (global longitudinal strain) or separately for basal, mid-cavity and apical segments (segmental longitudinal strain). **(C)** Representative longitudinal strain (orange dotted line) and -strain rate (blue solid line) curves along the endocardial border during three consecutive cardiac cycles. LS indicates longitudinal peak systolic strain. Peak strain rate was assessed during diastole (SR_*dia*_) and systole (SR_*sys*_), respectively. The interval between two R waves was defined as one cardiac cycle (corresponding ECG tracing is displayed on top). **(D)** Analysis of endocardial longitudinal strain. **(E)** Analysis of endocardial longitudinal SR_*sys*_. **(F)** Analysis of epicardial longitudinal strain. **(G)** Analysis of epicardial longitudinal SR_*sys*_. **(H)** Analysis of longitudinal SR_*dia*_. Mean ± SD or median and quartiles with 95% confidence interval; *n* = 10–11 per group; unpaired Student’s *t*-test or Mann-Whitney *U* test.

Longitudinal deformation was assessed along the endocardial and epicardial border on segmental level (base, mid-cavity, and apex) and globally as an average of the different segments (Figures 3A,B). Representative strain and strain rate curves are illustrated in Figure 3C. Global longitudinal strain along the endocardial border was significantly impaired in the HFD group, which was driven mainly by reduced basal strain values (Figure 3D). Global longitudinal SR_*sys*_ along the endocardium did not differ significantly (Figure 3E). Global longitudinal strain was also reduced along the epicardial border with significant impairment of basal and mid-cavity segments (Figure 3F). Global longitudinal SR_*sys*_ along the epicardium was significantly reduced in obese mice (Figure 3G). Similarly, the HFD group showed lower longitudinal SR_*dia*_ (Figure 3H). Radial and circumferential deformation remained preserved (Table 2).

### Myocardial Diffusion Metrics

**FIGURE 4:**
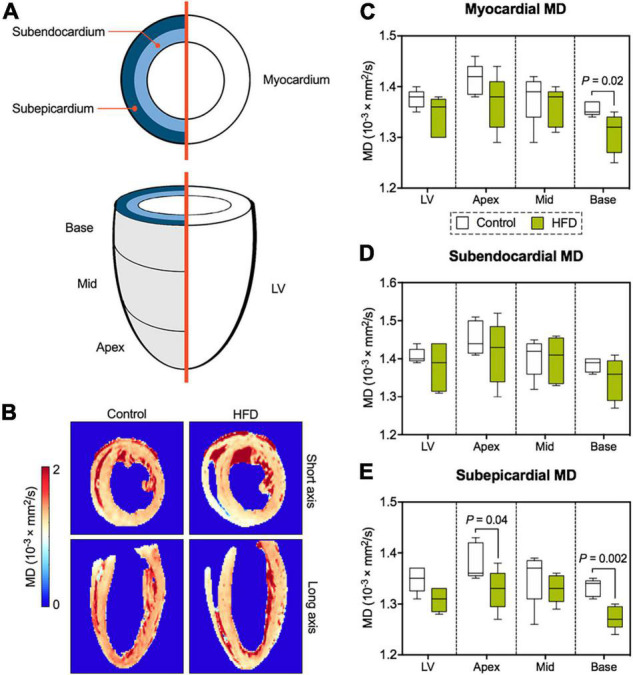
Analysis of myocardial diffusion metrics. **(A)** Illustration of DT-MRI segmentation. All DT-MRI parameters were assessed for both the global myocardium or separately for the subendocardial and subepicardial layer as well for the entire LV and its different segments (base/mid-cavity/apex). **(B)** Representative MD maps. Analysis of MD in the global myocardium **(C)**, the subendocardium **(D)**, and subepicardium **(E)** of the LV. Median and quartiles with 95% confidence interval; unpaired Student’s *t*-test; *n* = 5 per group.

Segmentation of the entire LV enabled comprehensive assessment of DT-MRI parameters with respect to myocardial layers (global myocardium vs. subendocardium/subepicardium) and segments (global LV vs. base/mid-cavity/apex) (Figure 4A). Myocardial mean diffusivity (MD) was significantly lower at basal level in obese animals (Figures 4B,C). While MD was comparable in the subendocardial layer, there was a significant drop in MD values in basal and apical segments of the subepicardium (Figures 4D,E).

**FIGURE 5:**
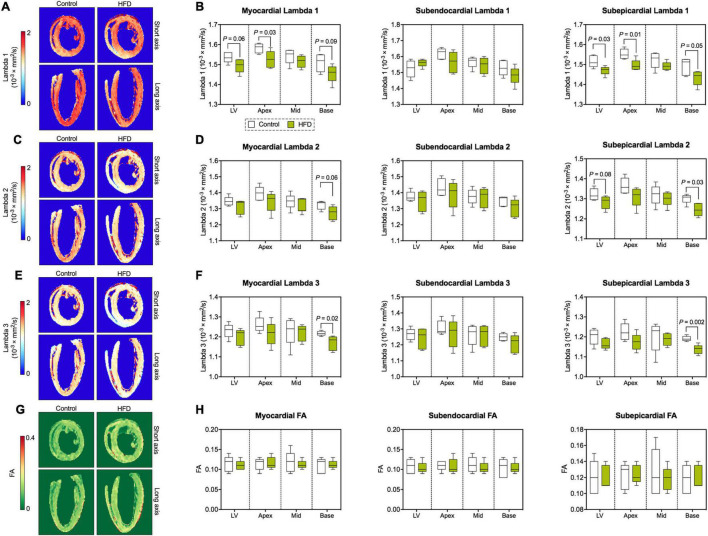
Eigenvalues of the diffusion tensor and FA. **(A)** Representative maps of the first eigenvalue of the diffusion tensor (lambda 1). **(B)** Analysis of lambda 1 (largest eigenvalue). **(C)** Representative maps of the second eigenvalue of the diffusion tensor (lambda 2). **(D)** Analysis of lambda 2. **(E)** Representative maps of the third (smallest) eigenvalue of the diffusion tensor (lambda 3). **(F)** Analysis of lambda 3. **(G)** Representative FA maps. **(H)** Analysis of FA. Median and quartiles with 95% confidence interval; *n* = 5 per group; unpaired Student’s *t*-test or Mann-Whitney *U* test.

Similar to MD, the magnitude of the main eigenvalue of the diffusion tensor (lambda 1) was lower in the entire myocardium, where significant differences were predominantly located in the basal and apical subepicardium (Figures 5A,B). Reductions in the second (Figures 5C,D) and third (Figures 5E,F) eigenvalue of the diffusion tensor were confined to epicardial layers, but also affected average values in the entire respective segment. While both MD and eigenvalues were reduced in the HFD group, the ratios between the different eigenvalues and, therefore, fractional anisotropy (FA) remained consistent with the control group (Figures 5G,H).

### Left-Ventricular Microstructure

**FIGURE 6:**
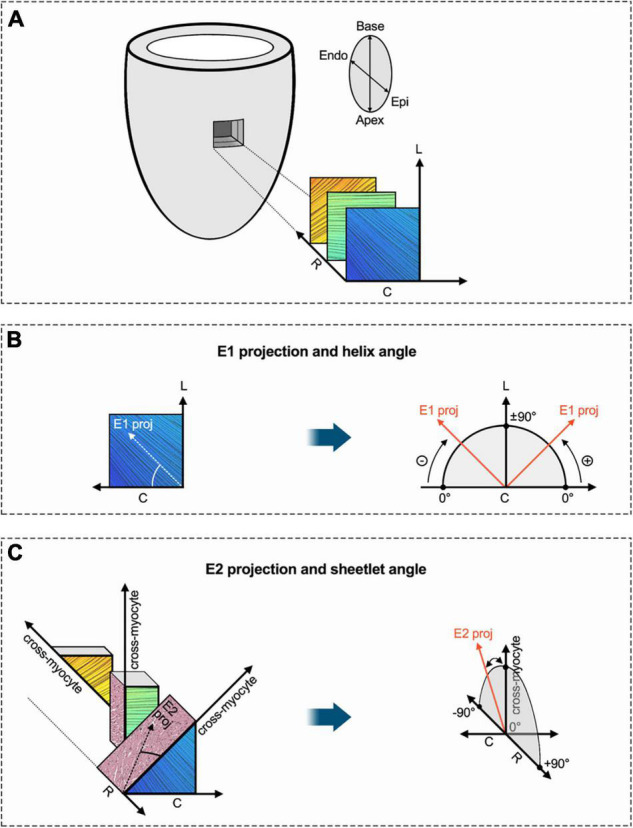
Schematic diagram of helix angle and sheetlet angle. **(A)** Transmural region of interest of the LV with exemplary visualization of subendocardial, mesocardial and subepicardial layers (fiber bundle excerpts are based on tractography in respective myocardial layers). In order to determine established DT-MRI metrics such as helix and sheetlet angle, a local coordinate system with circumferential, longitudinal, and radial axes is established for every voxel. **(B)** The primary eigenvector of the diffusion tensor (E1) was projected into the local tangential plane (given by local circumferential and longitudinal axes). The angle between E1 projection and the circumferential axis provides the so-called helix angle. As depicted in panel **(A)**, myofibers in the LV are arranged in two counter-directional helices with progressively changing pitch (positive in the subendocardium over neutral in the mesocardium to negative in the subepicardium). **(C)** The local cross-myocyte plane (given by local cross-myocyte orientation and radial axis) was determined perpendicular to the local primary eigenvector orientation (myofiber bundle orientation). A projection of the secondary eigenvector (E2) was placed in this cross-myocyte plane in order calculate the sheetlet angle E2A, which is defined as the angle between cross-myocyte direction and E2 projection. The metric aims to assess the impact of sheetlet and shear layer microstructure on diffusion components. Various cross-myocyte directions at different transmural levels (subepicardial, mesocardial, subendocardial) are depicted. Adapted from Ferreira et al. (34). C, circumferential; Endo, endocardium; Epi, epicardium; E1, primary eigenvector of the diffusion tensor; E2, secondary eigenvector of the diffusion tensor; L, longitudinal; R, radial.

**FIGURE 7:**
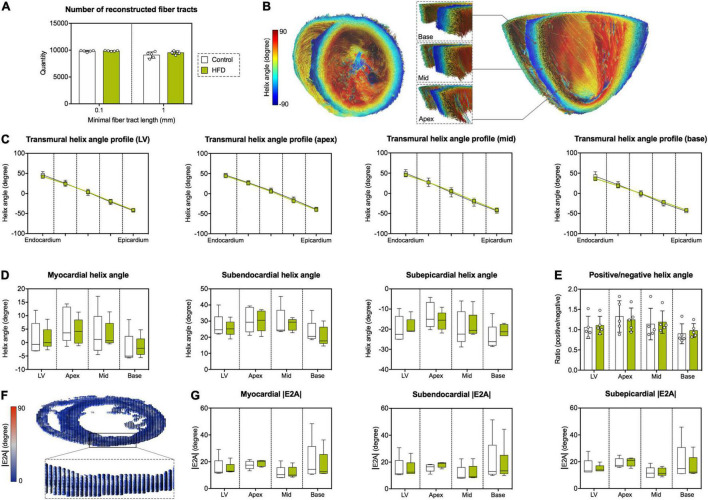
Assessment of left-ventricular microstructure by DT-MRI. **(A)** Number of reconstructed fiber tracts according to minimal fiber tract length as termination criterion (0.1 and 1 mm, respectively). **(B)** Representative reconstruction of cardiac fiber tracts with color-coded helix angle in an animal of the HFD group. Left: transverse section of the heart with a view in the direction of the apex. Right: longitudinal section of the heart in anterior-posterior direction with magnified regions of interest at basal, mid-cavity and apical level of the interventricular septum. **(C)** Transmural helix angle profiles in the LV and different myocardial segments. **(D)** Helix angles values in the different myocardial layers and segments. **(E)** Ratio of fiber tracts with a positive helix angle to fiber tracts with a negative helix angle in the different myocardial segments. **(F)** Visualization of the diffusion tensor in the cardiac short axis at mid-cavity level using superquadric glyphs. Color-coding corresponds to absolute sheetlet angle values (|E2A|). **(G)** Analysis of |E2A| in the different myocardial layers and segments. Mean ± SD **(C,E)** or median and quartiles with 95% confidence interval **(D,G)**; *n* = 5 per group.

Derived microstructure metrics were analyzed in the same fashion as diffusion metrics. The concept of myofiber tract geometry is illustrated in Figure 6. The number of reconstructed fiber tracts did not differ significantly between both groups (Figure 7A). Both groups displayed the stereotypical counter directional helical configuration of myocyte bundle tracts, exhibiting a smooth transmural progression from positive helix angle values in the subendocardium to negative helix angle values in the subepicardium (Figure 7B). No significant differences were found in any layer or segment for helix angle values or the transmural helix angle profile from endocardium to epicardium (Figures 7C,D). The relative proportion of fiber tracts with a positive helix angle were comparable between both groups (Figure 7E). In addition, we found no significant change in absolute angulation of the projection of the second eigenvector of the diffusion tensor (|E2A|) that is associated with sheetlet angulation (Figures 7F,G).

## Discussion

In this experimental study we demonstrated that (1) diet-induced obesity led to diastolic dysfunction and impaired longitudinal deformation; and (2) obesity-related cardiac dysfunction was association with altered cardiac diffusion properties in certain regions of the LV, whereas the three-dimensional myofiber arrangement remained preserved. In summary, the model of diet-induced obesity resembled several characteristics of obese patients at pre-heart failure states (corresponding to ACCF/AHA stages A and B), and myocardial microstructure was characterized by DT-MRI for the first time in this context.

Cardiovascular diseases are major determinants of the high morbidity and mortality of obesity, a condition whose global prevalence nearly tripled during the past ∼50 years, now reaching pandemic proportions (2, 3). Obesity is an independent risk factor for HF, with a “dose-response” relationship between body mass index and HF incidence in overweight and obese individuals (1). Several studies indicated that the deleterious effects of excessive body fat accumulation on the heart can be effectively reversed by weight loss (14). Therefore, identification of cardiac structural and functional abnormalities preceding overt HF is key to identify subjects at risk and to guide therapeutic interventions accordingly.

There is no uniform definition of obesity-related cardiac dysfunction, and cardiac changes in response to excessive body fat accumulation are quite heterogeneous (6). Here, we applied a well-established mouse model of diet-induced obesity reflecting the human situation of an unhealthy, obesity-prone lifestyle with gradual weight gain during adulthood. Similar to clinical observations, obese mice had diastolic dysfunction, reduced longitudinal strain and -strain rate as well as increased E/e’ ratios without affection of systolic pump function. Yet, fifteen weeks of HFD did not result in relevant LV wall-thickening, cardiomyocyte hypertrophy or myocardial fibrosis, contrasting several studies which show that long-term obesity can be associated with these characteristics in patients (6). As a likely explanation, the chosen HFD intervention for 15 weeks - especially in comparison to genetic models - yielded a more subtle obesity phenotype regarding long-term end organ damage [as reviewed previously (15)]. Therefore, future studies on myocardial microstructure in more advanced stages of obesity are required, especially to assess the impact of LV hypertrophy in this setting.

The present findings suggest that obesity is associated with alterations of cardiac diffusion properties, whose assessment may serve as a diagnostic marker in the future. Notably, these changes were detectable even in the absence of relevant cardiac hypertrophy, signs of overt HF, or histopathological evidence of myocardial damage. This implies that myocardial remodeling in response to excessive body fat accumulation may be present even in early disease stages, and that DT-MRI is capable of assessing these. We did not observe any changes of the three-dimensional myofiber- and sheetlet arrangement in the LV, which are major determinants of its mechanical and electrophysiological function. Future clinical studies evaluating DT-MRI in obese patients are warranted for the translation of our results.

Comprehensive image segmentation allowed the investigation of DT-MRI and myocardial strain parameters with respect to myocardial layers and segments. Diffusion in the myocardium is modulated by its multiple components such as cells, permeable membranes, and extracellular space (16). We observed a reduction of MD and the different eigenvalues of the diffusion tensor, which were found predominantly in the basal subepicardium. In general, MD is considered to decline with increased cellularity or reduced extracellular space, respectively (e.g., in case of cardiomyocyte hypertrophy/swelling and consecutive shrinkage of the extracellular compartment). Notably, histopathology indicated no relevant myocardial damage or cellular hypertrophy as a structural correlate in the present study. Our previous data showed that 15 weeks of HFD feeding induces cardiac lipid accumulation in mice (17). Therefore, the lower diffusivity in fat as compared to muscle tissue is likely to represent a structural correlate of reduced myocardial diffusivity in the used mouse model (18). This is supported by observations from Duchenne muscular dystrophy, where increased muscle fat content correlates with lower MD (19). Furthermore, fat accumulation in muscle tissue results in lower MD along all spatial directions (20), which is in accordance with our observation of a reduction in all three eigenvalues of the diffusion tensor.

There is mounting evidence that longitudinal deformation is impaired in obese individuals (6–9). Complementing these findings, obese mice showed abnormal longitudinal deformation predominantly in basal segments of the LV, which consistently demonstrated alterations in diffusion properties, too. Although clinical data on segmental differences in obesity-related cardiac dysfunction is scarce, previous studies have described a basal injury pattern in conditions related to inflammatory or systemic stressors like myocarditis (21, 22), cardiac amyloidosis (23) and Anderson-Fabry disease (24). Interestingly, a recent study reported on a similar echocardiographic pattern in hospitalized patients with coronavirus disease-19 and hypothesized that the susceptibility of the basal myocardium may be linked to epicardial adipose tissue (25).

Epicardial adipose tissue is located between the myocardium and the epicardium (visceral serous pericardium) without fascial separation, which is why it can be found directly within the subepicardial layer of the myocardium (26). The distribution of epicardial fat is asymmetric in humans and most abundant in the basal interventricular groove and the basally located coronary sulcus (27). Increased epicardial adipose tissue volume is associated with myocardial fat accumulation and reduced longitudinal deformation in obese subjects (8). Hence, it appears likely that our observation of reduced diffusivity and myocardial deformation in the basal subepicardium may be the result of increased epicardial fat content in this location. Nevertheless, fat quantity and -distribution were not assessed in the present study, and further research is essential to identify structural correlates of these novel findings in obesity-related cardiac dysfunction.

The vast majority of patients suffering from HFpEF are overweight or obese, and approximately 30 kg/m^2^ is the average body mass index reported in current HFpEF trials (28, 29). Indeed, obesity and corresponding cardiometabolic traits confer a higher risk of HFpEF than other forms of HF, and obesity-related HFpEF is considered a genuine form of cardiac failure (5, 30). Interestingly, the induction of arterial hypertension in addition to the used HFD protocol has previously been introduced as a new mouse model for HFpEF (31, 32). Given recent advances in clinical application of DT-MRI (16), we anticipate novel pathophysiological insights and diagnostic measures using this emerging imaging modality in patients with obesity-related cardiac dysfunction and HFpEF.

This study has several limitations that should be acknowledged. First, only a relatively small sample size was studied by DT-MRI due to long acquisition times and the complex post-processing. As the focus of this work was on myocardial microstructure, epicardial adipose tissue was removed during sample preparation, and its impact on cardiac diffusion properties remains to be examined. The small size of the murine heart limits layer-specific strain analysis to the endocardial and epicardial borders, which differs from clinical approaches for the assessment of myocardial layer-specific strain. In addition, the present study lacks a validated structural correlate for the observed decrease in diffusivity. Therefore, our results should be considered hypothesis-generating, and may serve as a pilot for future investigations. Fixation with formalin affects diffusion properties, which should be considered when comparing absolute diffusion metrics. Although diet-induced obesity generally does not induce type 2 diabetes in mice, the cardiac phenotype may be confounded by a mild glucose intolerance as shown before for BL/6 mice (33).

## Conclusion

Mice with diet-induced obesity displayed several features of patients with obesity-related cardiac dysfunction. DT-MRI revealed altered myocardial diffusion properties in this model, whereas the three-dimensional myofiber arrangement of the LV remained preserved. A regional pattern of reduced longitudinal strain and lower mean diffusivity in basal segments of the LV was newly identified. These findings indicate that DT-MRI may serve as a novel diagnostic tool in obesity-related cardiac dysfunction, and future studies are warranted for the translation of our results.

## Data Availability Statement

The raw data supporting the conclusions of this article will be made available by the authors, without undue reservation.

## Ethics Statement

The animal study was reviewed and approved by Landesamt für Gesundheit und Soziales Berlin, Germany.

## Author Contributions

DL: DT-MRI analyses, data acquisition, preparation and analysis, and writing. AT: animal study, echocardiography, data acquisition, preparation and analysis, and review and editing. MS: blood pressure measurements and echocardiographic analyses. VB: quantitative PCR, histopathology, review, and editing. ES: animal study. JS: review and editing. SB: data analysis, review, and editing. RK: histopathology. AF-L: conceptualization, project administration, and supervision. LS and UK: conceptualization, project administration, supervision, and funding acquisition. NB: blood pressure measurements, echocardiographic analyses, data acquisition, preparation and analysis, writing, conceptualization, project administration, and supervision. All authors read and revised the manuscript.

## Conflict of Interest

The authors declare that the research was conducted in the absence of any commercial or financial relationships that could be construed as a potential conflict of interest.

## Publisher’s Note

All claims expressed in this article are solely those of the authors and do not necessarily represent those of their affiliated organizations, or those of the publisher, the editors and the reviewers. Any product that may be evaluated in this article, or claim that may be made by its manufacturer, is not guaranteed or endorsed by the publisher.

## Footnotes

1
http://dsi-studio.labsolver.org
