# Supplementary material for: Assessment of Myocardial Microstructure in a Murine Model of Obesity-Related Cardiac Dysfunction by Diffusion Tensor Magnetic Resonance Imaging at 7T
Source: Front Cardiovasc Med. 2022 Apr 5;9:839714. doi: 10.3389/fcvm.2022.839714 (PMC9016133; doi:10.3389/fcvm.2022.839714)
Supplement: Supplementary file 1 [file Table_1.docx]

**Supplemental material**

| **Gene** | **Abbreviation** | **Forward primer (5'-3')** | **Reverse primer (5'-3')** |
| --- | --- | --- | --- |
| Atrial Natriuretic Peptide | *ANP* | CTGCTAGACCACCTGGAGGA | AAGCTGTTGCAGCCTAGTCC |
| Collagen Type I, Alpha 1 Chain | *Col1a1* | CTGACGCATGGCCAAGAAGA | ATACCTCGGGTTTCCACGTC |
| Collagen Type III, Alpha 1 Chain | *Col3a1* | CTGGTCCTGCTGGAAAGGAT | TCCATTGCGTCCATCAAAGC |
| Galectin-3 | *Gal3* | GCTTATCCTGGCTCAACTG | TTCACTGTGCCCATGATTGT |
| Beta-actin | *β-Actin* | GACAGGATGCAGAAGGAGATTACTG | GCTGATCCACATCTGCTGGAA |

**Supplemental Table 1:** Primer sequences used for quantitative real-time PCR.
